# Melanotic oncocytic metaplasia of the nasopharynx as a benign mimicker of malignant melanoma: a case report

**DOI:** 10.1186/1746-1596-5-5

**Published:** 2010-01-14

**Authors:** Takeshi Kondo, Kiyoshi Mori, Shiori Oka, Setsuko Morinaka

**Affiliations:** 1 Division of Pathology, Kobe University Graduate School of Medicine, Kobe, Japan; 2 Oka ENT Clinic, Kobe, Japan; 3 Department of Otorhinolaryngology, Kobe Japanpost Hospital, Kobe, Japan

## Abstract

**Introduction:**

Melanotic variant of oncocytic metaplasia of the nasopharynx is an extremely rare condition.

**Case report:**

A 73-year-old Japanese man presented with nasal congestion and chill. Nasoscopic examination revealed multiple black nodules around the bilateral torus tubarius. The nodules were biopsied to determine the histology. The clinical differential diagnosis was malignant melanoma or hemangioma. Microscopically, there were oncocytic plump cells with abundant brown pigmented granules showing glandular pattern. No significant atypia was found. The pigment was positive for Fontana-Masson staining, and negative for Berlin blue staining, showing that it was melanin pigment. Immunohistochemically, S100-positive HMB45-negative dendritic cells were also found.

**Conclusion:**

Such a pigmented variant of benign oncocytic lesion is very rare, and only 15 cases have been reported in the English literature. As a benign mimicker of malignant melanoma, melanocytic oncocytic metaplasia should be always taken into consideration in the clinical setting.

## Background

In 1995, Shek et al. originally reported two cases of melanotic oncocytic metaplasia of the nasopharynx [1]. Since then, only 13 cases have been reported in English literature [2-5]. We present here an additional case (the 16th case in English literature) of melanotic oncocytic metaplasia in the nasopharynx.

## Case presentation

**Figure 1:**
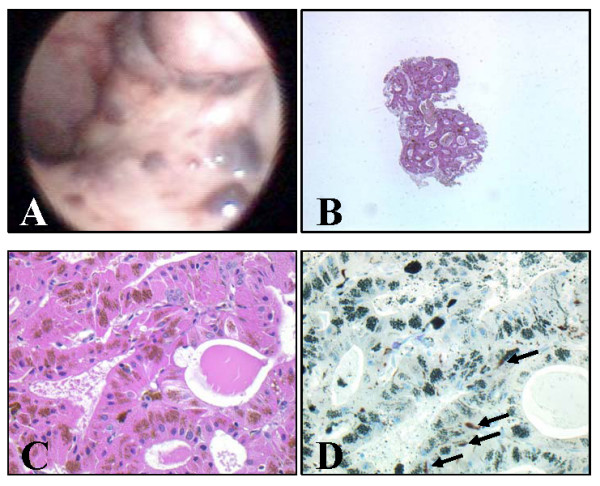
**Macroscopic and microscopic findings of the lesion**. A: Endonasal view of the nasopharynx, with multiple melanotic lesions around the Eustachian tuber (left side). B: Microscopic findings of the lesion (hematoxylin and eosin stain, low magnification). C: Microscopic findings of the lesion (hematoxylin and eosin stain, high magnification). Plump epithelial cells with eosinophilic cytoplasm and many brown pigmented granules are arranged in a tubular pattern. D: Immunohistochemical findings of the lesion (S-100). S100-positive HMB45-negative dendritic cells were scattered in the lesion (arrows). By counterstaining with Giemsa stain, melanin pigment showed heterochromasia (green).

A 73-year-old Japanese man presented with the feeling of nasal congestion (obstruction) and chill. His past medical history was unremarkable and he was a non-smoker. During nasoscopic examination multiple black nodules, measuring several millimeters, were discovered around the bilateral torus tubarius (Fig 1AB). While the patient was under followup at Oka ENT clinic for 4 months, the lesion grew slightly larger. The patient was then referred to Kobe Japanpost Hospital. No obstruction of the Eustachian tube opening was observed. His external auditory canal and tympanic membrane were normal. Examination of the neck, nasal cavity, and larynx revealed no abnormality. The clinical impression was that of malignant melanoma or hemangioma. This lesion was biopsied and histological examination was performed. Microscopically, the nodule was composed of plump epithelial cells with diffuse oncocytic metaplasia. These oncocytic cells had uniformly abundant eosinophilic granular cytoplasm on hematoxylin and eosin staining, and were arranged in a tubular or glandular pattern (Fig. 1C). Scattered brown pigments were also noted in the cytoplasm of oncocytic cells (Fig. 1C). Fontana-Masson stain confirmed that these pigments were melanin granules (not shown) and staining for hemosiderin (Berlin blue stain) was negative (not shown). By immunohistochemistry, S100-positive HMB45-negative dendritic cells were scattered in the lesion (Fig. 1D, arrows). Although oncocytic metaplasia occurring in melanoma has been reported [6], this lesion was easily differentiated from melanoma because of the absence of malignant component. Based on the above findings, the lesion was diagnosed as melanotic oncocytic metaplasia. The patient is doing well and the follow-up has been uneventful.

## Discussion

Oncocytic cells are large epithelial cells with eosinophilic, granular cytoplasm. Oncocytic metaplasia (change) is most frequently encountered in certain epithelial organs, such as the salivary gland, the lacrimal gland, the parathyroid gland, the thyroid gland, the pulmonary tree, and the kidney [4]. Oncocytes are not found in these organs of younger persons. The frequency of this oncocytic metaplastic change increases with age, its exact significance and biological function are, however, still unknown [2]. It is generally believed that the oncocyte represents a form of cellular degeneration [7]. Because oncocytes are discovered predominantly in elderly persons, they have undergone cytoplasmic change and are considered to be involved in an aging process [4].

Oncocytic change in the upper respiratory tract is an uncommon finding and melanotic variant of oncocytic metaplasia of the nasopharynx is an extremely rare condition. To our knowledge, 15 cases have been reported in the literature and our case is the 16th case.

The lesion may be single or multiple. Most of the lesions are asymptomatic and tend to be incidentally discovered during examination of the nasopharynx. Some of these lesions may occasionally produce symptoms. When the mucosa around the opening of Eustachian tube is affected, ear or nose symptoms may occur. Eustachian tube dysfunction can be caused when the tissue edema accompanying the lesions impairs the tube function. In this case, the feeling of nasal congestion (obstruction) may be due to Eustachian tube dysfunction, and the chill may be coincidental.

All reported cases including our case are elderly males in their sixties or seventies. The lesion may be misinterpreted clinically as early nasopharyngeal carcinoma, nevus, or malignant melanoma. The origin of the melanin pigment is in question. The exact nature of melanin-containing oncocytes in the nasopharynx awaits further clarification.

## Conclusion

In conclusion, we report the 16th case of melanin-containing oncocytic metaplasia of the nasopharynx. As a benign mimicker of malignant melanoma, melanocytic oncocytic metaplasia should be always taken into consideration in the clinical setting.

## Consent

Written informed consent was obtained from the patient for publication of this case report and accompanying images. A copy of the written consent is available for review by the Editor-in-Chief of this journal.

## Competing interests

The authors declare that they have no competing interests.

## Authors' contributions

TK and KM performed histological examination and analyzed the case. TK was a major contributor in writing the manuscript. SO and SM treated the patient and provided clinical images. All authors read and approved the final manuscript.
